# Allaitement maternel et diversification alimentaire à Lubumbashi (République Démocratique du Congo): besoin urgent d’éducation des mères pour le changement des habitudes

**DOI:** 10.11604/pamj.2013.14.142.2436

**Published:** 2013-04-10

**Authors:** Toni Kasole Lubala, Augustin Mulangu Mutombo, Adonis Muganza Nyenga, Paul Ilunga Makinko, Gray a Wakamb Kanteng, Félix Kitengewa Momat

**Affiliations:** 1 Département de Pédiatrie, Faculté de Médecine, Université de Lubumbashi, République Démocratique du Congo; 2 Département de Gynécologie-Obstétrique-Faculté de Médecine-Université de Lubumbashi, République Démocratique du Congo

**Keywords:** Allaitement maternel, diversification alimentaire, République Démocratique du Congo, breast-feeding, dietary diversification, Democratic Republic of Congo

## Aux éditeurs du Journal Panafricain de Médecine

Les habitudes d'alimentation du nourrisson Africain sont influencées par de nombreuses considérations socio-culturelles [1]. Dans certains cas, celles-ci peuvent être à la base de graves troubles nutritionnels aigus mettant en jeu le pronostic vital.

En République Démocratique du Congo, la malnutrition est encore à l'heure actuelle l'un des problèmes nutritionnels majeurs chez les enfants de moins de 2 ans. En effet, ici, comme ailleurs dans les pays à faibles revenus, plus de la moitié de la mortalité infantile est associée aux maladies nutritionnelles [2]. L'atteinte de l'objectif 4 du millénaire visant la réduction de la mortalité infantile de 2/3 d'ici 2015 passe donc obligatoirement par la prévention des maladies nutritionnelles survenant dans les premières années de la vie.

Nous avons interrogé 150 mères d'enfants âgés de 2 à 5 ans au sujet de leurs habitudes d'alimentation. Leur moyenne d’âge était de 27 ± 5 ans et celle des enfants de 3.5 ± 1 an. Près du quart des enfants présentait une malnutrition modérée (Z-score poids pour âge (-2 et -1), au moment de l'enquête.

Nous avons constaté que 30.67% des mères ont allaité exclusivement jusqu’à 6 mois. Ce taux est inférieur aux valeurs nationales (36%) [3], Sénégalaises (34) [4] et mondiales (40%). Pourtant, une large étude randomisée a permis de montrer que l'allaitement maternel exclusif et prolongé permet de réduire l'incidence des infections gastro intestinales et des eczémas atopiques dans la première année de vie [5, 6]. De plus, un allaitement exclusif et prolongé améliore de 5.9 l’échelle globale du quotient intellectuel [7]. Il existe donc une évidence sur les bénéfices de l'allaitement maternel exclusif pour l'enfant. Un travail important devrait donc être abattu pour promouvoir davantage l'allaitement maternel exclusif jusqu’à 6 mois à Lubumbashi.

Dans notre série, 69.33% des mères interrogées n'ont pas allaité exclusivement principalement pour des pleurs récurrents perçus comme une absence de satiété liée à une insuffisance de production de lait. La croyance selon laquelle beaucoup de mères ne sont pas capables de produire assez de lait est profondément enracinée et extrêmement répandue dans la population et semble être culturellement construite. Pourtant, dans bien des cas, cette impression est subjective.

L'allaitement maternel était perçu par de nombreuses mères comme une cause d'altération de la morphologie des seins. L'indisponibilité de la mère pour raisons professionnelles a aussi été évoquée.

L’âge moyen d'introduction de l'allaitement mixte était de 3.37 ± 0.855 mois et l’âge moyen du sevrage de 9.19 ± 4 mois. Lorsqu'on s'intéresse au type de lait introduit, nous constatons que dans 87% des cas, il s'agissait d'un lait de vache entier. Dans 36.67% des cas la diversification alimentaire a été réalisée avec des aliments de transition inadaptés (à base de farine de maïs et farine de froment). Ces habitudes d'alimentation exposent les enfants à des manifestations allergiques de type eczema et wheezing récurrent [8]. De plus, la consommation avant 6 mois de farines contenant du gluten expose au risque d'apparition d'une maladie cœliaque cliniquement symptomatique ou silencieuse sur les terrains génétiquement prédisposés [9].

## Conclusion

A Lubumbashi, en République Démocratique du Congo, nous avons constaté un faible taux d'allaitement exclusif jusqu’à 6 mois et l'introduction précoce de laits de vache dans l'alimentation des nourrissons. Par ailleurs, la diversification alimentaire est précoce et les aliments de diversification inadaptés dans la majorité des cas. Il existe donc de toute évidence un besoin urgent d’éducation des mères sur les recommandations en matière d'allaitement, de sevrage et de diversification alimentaire à Lubumbashi. Cette éducation pourrait être intensifiée dans le cadre des activités de protection maternelle et infantile (Consultations prénatales et consultations préscolaires) prévues dans le paquet des soins de santé primaire en République Démocratique du Congo.
